# Epithelial-Mesenchymal Transition and Somatic Alteration in Colorectal Cancer with and without Peritoneal Carcinomatosis

**DOI:** 10.1155/2014/629496

**Published:** 2014-08-03

**Authors:** Y. A. Shelygin, N. I. Pospekhova, V. P. Shubin, V. N. Kashnikov, S. A. Frolov, O. I. Sushkov, S. I. Achkasov, A. S. Tsukanov

**Affiliations:** State Scientific Centre of Coloproctology, 2 Salyam Adil Street, Moscow 123423, Russia

## Abstract

Colorectal cancer is highly metastatic even when the tumors are small. To disseminate, cells use a complex and multistage process known as the epithelial-mesenchymal transition, in which epithelial phenotype is transformed into mesenchymal phenotype. The objective of this study is to describe the epithelial-mesenchymal transition in terms of gene expression profile and somatic alterations in samples of colorectal cancer with or without peritoneal carcinomatosis. We analyzed samples taken from 38 patients with colorectal cancer (stages II-IV) and samples from 20 patients with colorectal cancer complicated by peritoneal carcinomatosis. The expression of *ZEB1, ZEB2, CDH1, VIM*, and *SNAI1* was analyzed by real-time PCR. *KRAS/BRAF* mutations were mapped using sequencing. Microsatellite instability was evaluated by fragment analysis. Epithelial-mesenchymal transition was detected in 6 out of 38 samples of colorectal cancer (stages II-IV), 7 out of 20 tumors from patients with peritoneal carcinomatosis, and 19 out of 20 samples taken from carcinomatous nodules. Tumors of the mesenchymal subtype displayed high frequency of somatic mutations, microsatellite stability, and low degree of differentiation. The identification of epithelial-mesenchymal transition may be used as a marker of high metastatic potential, which is particularly relevant at early stages of tumor growth.

## 1. Introduction

Adenocarcinomas originating in intestinal epithelium make up an overwhelming majority of malignant tumors of the colon. As the tumor grows, its cells infiltrate the surrounding stroma, penetrate blood and lymphatic vessels, and are passively carried to remote organs, where they form metastases. Such spreading of the primary tumor, or metastasis, is the leading cause of death from colorectal cancer (CRC). In 2002 the French oncologist Thiery formulated a hypothesis explaining how metastasis occurs [1]. To disseminate, tumor cells use a complex and multistage process, in which epithelial phenotype is transformed into mesenchymal phenotype. This process is now known as the epithelial-mesenchymal transition (EMT). The sequence of events characteristic of the EMT is crucial to the formation and differentiation of body organs during embryonal development. As a pathological process, EMT triggers tumor progression; its cells acquire migrating potential and may invade the surrounding stroma and enter circulating blood [1–3]. The expression of a considerable number of genes is altered during EMT; some transcription factors (*SNAI1/2, TWIST, ZEB1/2,* and so on) and mesenchymal markers are overexpressed, while the expression of epithelial phenotype markers is suppressed.

The objective of this study is to describe the epithelial-mesenchymal transition in terms of gene expression profile and somatic changes, molecular as well as genetic, in samples of colorectal cancer of various stages, with or without peritoneal carcinomatosis (PC).

## 2. Materials and Methods

### 2.1. Patients

This study was performed in samples of tumors, carcinomatous nodules, and healthy mucous membranes (in total 136 samples), which were obtained from the colon of 58 patients undergoing surgery for colorectal cancer at the State Research Center of Coloproctology between November 2012 and February 2014. In 38 cases we collected a sample from both the tumor and the normal mucosa, and in 20 cases three samples were taken from the tumor, carcinomatous nodulus, and normal mucosa. The clinical characteristics of all patients are listed in Table 1.

### 2.2. DNA and RNA Extraction

DNA was extracted from the tumors using a PROBE-GS-GENETICA kit (DNA Technology, Russia) according to the procedure described by the manufacturer. Tissue samples were placed in lysis buffer immediately after collection, and total RNA was extracted by PureLink RNA Mini Kit (Ambion, USA) following the procedure specified by the manufacturer. The quality of RNA extraction was verified by electrophoresis in 1.8% agarose gel. The product was stained with ethidium bromide and analyzed using Gel Doc XR+ imaging system (BioRad, USA) in ultraviolet light. The concentration of extracted RNA was measured with a P300 spectrophotometer (IMPLEN).

### 2.3. Detection of Mutations

Somatic mutations in the* KRAS* (exon 2, codons 12/13) and* BRAF* (codon 15, V600E) genes were detected by polymerase chain reaction and a Tertsik amplifier (DNA Technology, Russia), and both complementary chains were sequenced with ABI PRISM 3500 (8 capillaries; Applied Biosystems, USA).

### 2.4. Microsatellite Instability

Microsatellite instability was evaluated in tumor samples using fragment analysis for five markers (NR21, NR24, NR27, BAT25, and BAT26) with ABI PRISM 3500 (8 capillaries; Applied Biosystems, USA).

### 2.5. Reverse Transcription

Reverse transcription was performed with ImProm-II Reverse Transcriptase kit (Promega) in accordance with the procedure described by the manufacturer. Once the reaction was complete, we measured the concentration of cDNA with a P300 spectrophotometer (IMPLEN).

### 2.6. Real-Time PCR

To evaluate gene expression, we used StepOnePlus (Applied Biosystems, USA). PCR was performed with 20 *μ*L of solution consisting of: 100–200 ng of DNA, 10 pM of gene-specific primers, 2 mM dNTP, 0.5 U Taq DNA polymerase (Sib Enzyme, Russia), PCR buffer, and Eva Green dye. Ct values for each gene were normalized by Ct values for control genes. Two control genes were analyzed:* GAPDH* and* TFRC*. The change in gene expression was calculated using ΔΔCt method (estimated as lg).

### 2.7. Statistical Analysis

Statistical analysis was performed in standard Statistica software (version 10.0, Statsoft Inc., USA) using *χ* ^2^ and Fisher's exact test for four-cell tables.

## 3. Results

Gene expression was analyzed using real-time PCR in tumor samples obtained from patients (*n* = 58) with CRC of different types defined by TNM classification, morphological characteristics, and presence or absence of peritoneal carcinomatosis (PC). The epithelial-mesenchymal transition (EMT) program was analyzed with regard to the expression of five genes (*ZEB1, ZEB2, CDH1, VIM, *and* SNAI1*), which were selected based on previous studies and are known to be associated with progressive EMT. Once this process is under way in a tumor, it is typical to find a coordinated alteration in the expression of all these genes: the expression of* ZEB1, ZEB2, VIM,* and* SNAI1* is upregulated, while the expression of* CDH1* is downregulated.

### 3.1. EMT and Somatic Mutations in the* KRAS* and* BRAF* Genes and MSI Status in Stage II-IV Colorectal Cancer

**Figure 1:**
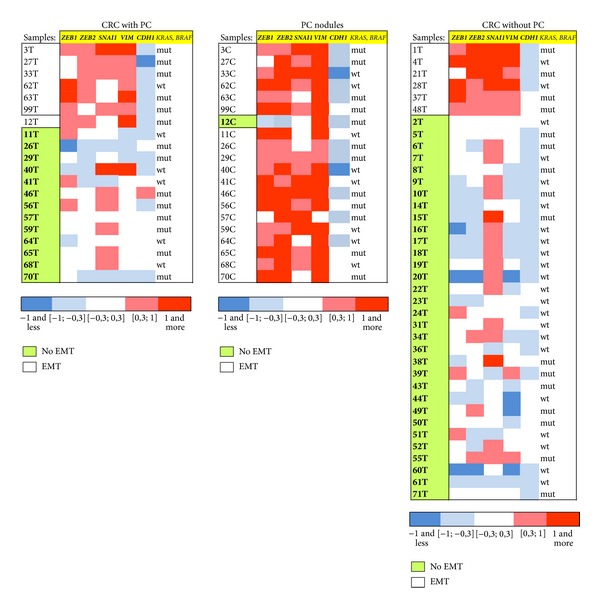
The gene expression signature in CRC without/with PC and carcinomatous nodules.

EMT process was detected in 6 out of 38 (15.8%) samples of CRC. The gene expression signature in samples with and without EMT is shown in Figure 1.

The frequency and characteristics of some molecular and genetic alterations typical for CRC, such as mutations in* KRAS* and* BRAF* genes and MSI status, are presented in Table 2.

Mutations in the* KRAS* gene were detected in 39.5% of tumors. V600E mutation was discovered in the* BRAF* gene of one patient. The majority of tumors were microsatellite stable—MSS (84.2%). A high level of microsatellite instability (MSI-Н) was detected in 5 samples.

### 3.2. EMT and Somatic Mutations in the* KRAS* and* BRAF* Genes and MSI Status in Colorectal Cancer with Peritoneal Carcinomatosis

We analyzed paired samples from the tumors/carcinomatous nodules of 20 patients with CRC and peritoneal carcinomatosis. EMT was detected in 7 out of 20 samples of the primary tumor (35%). However, the frequency of EMT reached 95% in carcinomatous nodules (19 out of 20). The gene expression signature in samples of primary tumors and carcinomatous nodules is shown in Figure 1.

The frequency and characteristics of somatic mutations in the* KRAS* and* BRAF* genes and MSI status are presented in Table 2.

Mutations in the* KRAS* gene were detected in 55% of all tumors. V600E-*BRAF* mutation was found in 3 samples. In two cases we discovered discordance between the primary tumor and the carcinomatous nodulus in terms of their mutational status: a mutation detected in the tumor was absent in the sample taken from the carcinomatous nodulus. However, the gene expression profile in carcinomatous nodules corresponded to the mesenchymal subtype. The overall frequency of mutations in both genes in CRC with PC was 70%, compared to 39% in CRC without carcinomatosis; this difference was statistically significant (*P* = 0.04). Eighteen samples taken from tumors were microsatellite stable (90%). High level of microsatellite instability was not detected in any of the analyzed samples.

### 3.3. Pathomorphological Characteristics of the Tumors

The frequency of highly, moderately, and poorly differentiated tumors among study samples is presented in Table 3. CRC with carcinomatosis was typically poorly differentiated compared to CRC without carcinomatosis (*P* = 0.006).

### 3.4. A Comparison of EMT-Negative and EMT-Positive Tumors

**Figure 2:**
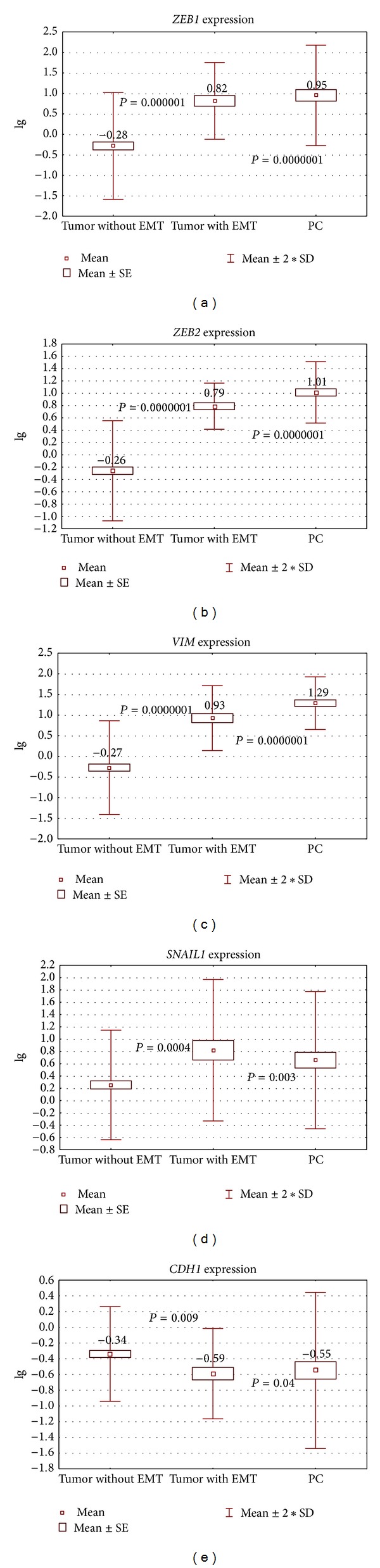
The expression levels of genes in samples with/without EMT and carcinomatous nodules.

EMT was detected in 13 out of the total of 58 tumor samples (22.4%) and in 19 out of 20 carcinomatous nodules (95%). The mean expression levels of* ZEB1, ZEB2, VIM, SNAI1, and CDH1* genes in samples with and without EMT and carcinomatous nodules are shown in Figure 2. The difference of expression levels of all genes between EMT-negative tumors/EMT-positive tumors and EMT-negative tumors/carcinomatous nodules was statistically significant.

The frequencies of somatic mutations in the* KRAS* and* BRAF* genes and MSI status in EMT-negative and EMT-positive tumors are presented in Table 4.

The* BRAF*-mutation frequency in EMT-negative CRC was 2.2%, compared to 23.1% in EMT-positive CRC; this difference was statistically significant (*P* = 0.03). The overall frequency of mutations in both genes in EMT-negative CRC was 44.4%, compared to 76.9% in EMT-positive CRC; this difference was statistically significant (*P* = 0.039).

The number of moderately and poorly differentiated tumors is shown in Table 5. EMT-positive tumors were usually poorly differentiated compared to EMT-negative tumors (*P* = 0.001).

## 4. Discussion

The current view is that EMT, which transforms immobile epithelial cells into mobile and invasive cells, plays a central role in enhancing the metastatic potential of various cancers, including CRC. This multistage process is accompanied by structural and morphological alterations in tumor cells, whose morphology is transformed as a result [1–3].

In recent studies CRC was subdivided into various molecular subtypes depending on gene transcription profiles, somatic mutations, MSI status, and gene methylation status. In the genetic classification of CRC developed by Roepman et al. [4] this cancer is divided into three subtypes: A-subtype, B-subtype, and C-subtype. The molecular and genetic criteria employed in this classification include epithelial-mesenchymal transition, defect in the DNA mismatch-repair system manifested as a high degree of microsatellite instability, and proliferation activity of tumor cells. Marisa et al. distinguish among seven subtypes of CRC [5]. Another study by Zhu et al. describes three different subtypes [6]. However, in all these studies one of CRC subtypes is colorectal cancer associated with triggering the EMT program.

In our study EMT was detected in samples of colorectal cancer based on coordinated changes in the expression of the following genes:* ZEB1*,* ZEB2*,* SNAI1*,* CDH1*, and* VIM*. The expression of these genes was studied because IHC analysis was not possible to carry out technically. The protein products of* ZEB1*,* ZEB2*, and* SNAIL1* are transcription factors which, along with some other factors, play a key role in triggering the EMT [7–9]. Proteins E-cadherin (encoded by the* CDH1* gene) and vimentin (encoded by* VIM*) are cellular markers of epithelial and mesenchymal tissues, respectively. The coordinated upregulation or, not uncommonly, overexpression of* ZEB1*,* ZEB2*,* SNAI1*, and* VIM* and the downregulation of* CDH1* indicated that EMT was under way, making the tumor cells assume a mesenchymal phenotype. Notably, overexpression of* SNAI1* and downregulation of* CDH1* were often observed in other samples, which we classified them as EMT-negative. This phenotype apparently reflects an ongoing transition and can be seen as an intermediate type between the epithelial and the mesenchymal subtypes of tumors [10]. Only 13 out of 58 (22.4%) primary tumors were judged to be EMT-positive, including 6 out of 38 cases of stage II-IV CRC and 7 out of 20 cases of CRC with peritoneal carcinomatosis. In many studies, such as that by Roepman et al. [4] mentioned above, the mesenchymal subtype of CRC is described as a tumor with intrinsically poor prognosis and resistant to adjuvant chemotherapy. In our sample, a considerable proportion of patients (*n* = 20) had CRC with peritoneal carcinomatosis. Cells from the primary tumor that have migrated into the peritoneum are the likely source of carcinomatous nodules. It is these cells, which assume a mesenchymal phenotype with overexpression of vimentin and suppressed expression of* CDH1*, leading to disrupted regulation of cellular adhesion, that may have been the source of metastases. All but one sample from peritoneal nodules (95%) were of the mesenchymal phenotype. In one case EMT was detected only in the tumor. EMT concordance was observed only in 6 cases, where the expression profile was nearly identical in the primary tumor and the peritoneal nodulus. Possibly, the cells of the primary tumor may lose their mesenchymal phenotype over time (a reverse epithelial-mesenchymal transition) [11], or else the tumor may be highly heterogeneous [12]. The fact that the EMT was detected in an overwhelming majority of carcinomatous tumors in this study thus proves that CRC of the mesenchymal subtype is aggressive and confers a poor prognosis. As for stage II-III tumors, the EMT was detected in 5 out of 35 samples (14.3%). This value is very close to that reported by Roeman et al. [4], who classified 16% of tumors as C-subtype (mesenchymal tumors).

The frequency of somatic mutations in the* KRAS* and* BRAF* genes varies in tumors of these two subtypes; but it is significantly higher in mesenchymal tumors, and 3 out of 4* BRAF*-V600E mutations were discovered in these tumors. According to Roepman et al. [4], CRC of the mesenchymal subtype abounds in mutations in these genes, especially in* BRAF*. Activating mutations in this gene are interpreted as a poor prognostic factor in CRC patients [13]. The association between these mutations and the mesenchymal phenotype was demonstrated by Makrodouli et al. [14]. The data they collected using cell lines of colon adenocarcinoma shows that* BRAF*-V600E induces migration and enhances the invasive potential of these cells. One of the mutations found in* KRAS* (G12V) also promotes cellular migration and invasion [14]. It is worth pointing out that we detected G12V among* KRAS* mutations on four occasions, of which three were in EMT-positive samples. All tumors with a high degree of microsatellite instability (MSI-H) were of the epithelial subtype, which is in line with the traditional interpretation of microsatellite instability in a tumor as a good prognostic factor.

To summarize, our study of the profile of gene expression identified the tumors undergoing the process of epithelial-mesenchymal transition. Classifying cancer cases based on EMT may help to detect the malignant mesenchymal subtype, which is associated with poorly differentiated and highly metastatic tumors. The association between EMT and peritoneal carcinomatosis indicates that EMT-positive tumors have high metastatic potential, which is particularly important at early stages of tumor growth.

## Figures and Tables

**Table 1:** The clinical characteristics of patients.

Characteristics	CRC without PC (*n* = 38)	CRC with PC (*n* = 20)
Mean age (min–max)	62.1 (32–81)	63.7 (26–84)
Male/female	13/25	11/9
Tumor localization:		
Rectum	4	1
Left side	21	8
Right side	13	11
Stage:		
I (T1-2N0M0)	2	
II (T3-4N0M0)	17	
III (TanyN1-2M0)	16	
IV (TanyNanyM1)	3	20

**Table 2:** The *KRAS*- and *BRAF*-mutation frequency and MSI status in CRC without and with PC.

	CRC without PC, *n* = 38	%	CRC with PC, *n* = 20	%	*P*
*KRAS*-mut	15	39.5	11	55	0.197
*BRAF* V600E	1	2.6	3	15	0.114
*KRAS*-mut + *BRAF* V600E	16	42.1	14	70	**0.04**
*KRAS* + *BRAF* wt	22	57.9	6	30	**0.04**
MSI-Н	5	13.2	0	0	0.11
MSI-L	1	2.6	2	10	0.23
MSS	32	84.2	18	90	0.43

**Table 3:** The data of tumor grade.

Grade	CRC without PC, *n* = 38 (%)	CRC with PC, *n* = 20 (%)	*P*
G1	0	0	
G2	24 (63.2)	6 (30)	**0.006 **
G3	11 (28.9)	14 (70)	**0.006**
Unknown	3	0	

**Table 4:** The *KRAS*- and *BRAF*-mutation frequency and MSI status in EMT-negative and EMT-positive tumors.

	EMT-negative CRC, *n* = 45 (%)	EMT-positive CRC, *n* = 13 (%)	*P*
*KRAS*-mut	19 (42.2)	7 (53.8%)	0.33
*BRAF* V600E	1 (2.2)	3 (23.1)	**0.03**
*KRAS*-mut + *BRAF* V600E	20 (44.4)	10 (76.9)	**0.039**
*KRAS* + *BRAF* wt	25 (55.6)	3 (23.1)	**0.039**
MSI-Н	5	0	0.27
MSI-L	2	1	0.54
MSS	38	12	0.42

**Table 5:** The data of EMT-negative/EMT-positive tumors grade.

Grade	EMT-negative CRC, *n* = 45 (%)	EMT-positive CRC, *n* = 13 (%)	*P*
G1	0	0	
G2	28 (62.2)	2 (15.4)	**0.001**
G3	14 (31.1)	11 (84.6)	**0.001**
Unknown	3	0	

## References

[1] Thiery JP (2002). Epithelial-mesenchymal transitions in tumour progression. *Nature Reviews Cancer*.

[2] Yang J, Weinberg RA (2008). Epithelial- mesenchymal transition: at the crossroads of development and tumor metastasis. *Developmental Cell*.

[3] Thiery JP, Acloque H, Huang RYJ, Nieto MA (2009). Epithelial-mesenchymal transitions in development and disease. *Cell*.

[4] Roepman P, Schlicker A, Tabernero J (2013). Colorectal cancer intrinsic subtypes predict chemotherapy benefit, deficient mismatch repair and epithelial-to-mesenchymal transition. *International Journal of Cancer*.

[5] Marisa L, de Reynies A, Duval A (2013). Gene expression classification of colon cancer into molecular subtypes: characterization, validation, and prognostic value. *PLOS Medicine*.

[6] Zhu J, Wang J, Shi Z (2013). Deciphering genomic alterations in colorectal cancer through transcriptional subtype-based network analysis. *PLoS One*.

[7] Peinado H, Olmeda D, Cano A (2007). Snail, ZEB and bHLH factors in tumour progression: an alliance against the epithelial phenotype?. *Nature Reviews Cancer*.

[8] Joyce T, Cantarella D, Isella C, Medico E, Pintzas A (2009). A molecular signature for Epithelial to Mesenchymal transition in a human colon cancer cell system is revealed by large-scale microarray analysis. *Clinical and Experimental Metastasis*.

[9] Lim J, Thiery JP (2012). Epithelial-mesenchymal transitions: insights from development. *Development*.

[10] Huang RY-J, Wong MK, Tan TZ (2013). An EMT spectrum defines an anoikis-resistant and spheroidogenic intermediate mesenchymal state that is sensitive to e-cadherin restoration by a src-kinase inhibitor, saracatinib (AZD0530). *Cell Death and Disease*.

[11] Sipos F, Galamb O (2012). Epithelial-to-mesenchymal and mesenchymal-to-epithelial transitions in the colon. *World Journal of Gastroenterology*.

[12] Meacham C, Morrison S (2013). Tumour heterogeneity and cancer cell plasticity. *Nature*.

[13] Souglakos J, Philips J, Wang R (2009). Prognostic and predictive value of common mutations for treatment response and survival in patients with metastatic colorectal cancer. *British Journal of Cancer*.

[14] Makrodouli E, Oikonomou E, Koc M (2011). BRAF and RAS oncogenes regulate Rho GTPase pathways to mediate migration and invasion properties in human colon cancer cells: a comparative study. *Molecular Cancer*.

